# Mental health during the coronavirus disease 2019 (Covid-19) pandemic: more is still to be done

**DOI:** 10.11604/pamj.2020.35.7.22605

**Published:** 2020-04-16

**Authors:** Francky Teddy Endomba, Dominic Leandry Angong Wouna, Celestin Danwang

**Affiliations:** 1 Psychiatry Internship Program, University of Bourgogne, 21000 Dijon, France; 2 Bertoua Regional Hospital, Bertoua, Cameroon; 3 Epidemiology and Biostatistics Unit, Institute of Experimental and Clinical Research, Université Catholique de Louvain, Brussels, Belgium

**Keywords:** Mental health, novel coronavirus, pandemic, psychological support

## To the editors of Pan African Medical Journal

Initially described as pneumonia of unknown aetiology in December 2019 in populations of Wuhan (China), the coronavirus disease (Covid-19) rapidly disseminated around the world [1]. On 11 March 2020, the World Health Organization (WHO) characterized Covid-19 as a pandemic [1]. Although the burden of Covid-19 is chiefly due to its rapid spread and harmful respiratory consequences, its impact on populations mental health is not to be neglected. The first persons undergoing psychological repercussions are suspected or confirmed Covid-19 patients. Indeed, aware of the disease mortality, they may experience various degrees of psychological distress linked to fear of death [2]. Outbreaks also affect mental health of healthy individuals. Wang and colleagues studied 1210 individuals from 194 cities in China during the initial stage of Covid-19 and found that the psychological impact was moderate to severe for 53.8% of them [3]. They also reported that respectively 16.5% and 28.8% experienced moderate to severe depressive symptoms and moderate to severe anxiety symptoms [3]. Moreover, among healthy persons, there is a risk of health anxiety which can results in maladaptive safety behaviours such as inappropriate self-medication [4].

Actually, nearly three billions people are confined at homes or in quarantine around the world. Brooks et al. in a recently published review including studies comparing psychological outcomes between people quarantined and not quarantined, found that quarantine had negative psychological effects such as post-traumatic stress symptoms, confusion and anger [5]. All these psychological consequences are more likely to be encountered among mental health patients. As for precedent outbreaks, healthcare workers are at the first line during this Covid-19 pandemic. In February 2020, a multicentre survey involving 1563 health workers in China found that 50.7% had depression, 44.7% had anxiety and 73.4% had stress-related symptoms [6]. The WHO developed mental health and psychological considerations at the time of the Covid-19 outbreak [7]. These considerations are tailored messages targeting different populations groups including general population, healthcare workers, team leaders or managers in health facility, care providers for children, older adults, care providers, people with underlying conditions and people in isolation [7]. Notwithstanding these general recommendations, various specific strategies have been developed by some countries in order to provide psychological help to populations. According to Xiang and colleagues, mental health care response involves three main factors: 1) mental health teams, 2) clear communication with regular and accurate updates and 3) secure services (notably using electronic devices) for psychological support [8]. These strategies could help countries in which the Covid-19 pandemic is at the beginning such as Sub-Saharan African ones. One other example of psychological intervention crisis that we can highlight is the one of Shanghai [9].

Shanghai government organized psychological care in four levels. At the first level, an onsite psychological support is provide to patients with severe symptoms of novel coronavirus pneumonia (NCP) by front-line medical staff, Centre for Disease Control researchers or administrative staff [9]. The second level concerns patients with mild symptoms of NCP, close contacts, suspected patients or patients with fever attending to hospital for treatment, and they also have onsite services [9]. The third level includes people related to the first and second ones such as family members, colleagues or friends, and rescuers while the fourth level encompasses people in affected areas, going from susceptible groups to general public [9]. For these two last levels, a 24/7 real-time remote (telephone and internet) psychological support is delivered [9]. Taking-to-apart the case of health-care workers mental health, synergistically to considerations elaborated by the WHO [7], it seems that more should be done especially in areas where the Covid-19 has greater morbidity and mortality, and leads to overwhelmed health systems. Indeed, in such areas, caregivers could be more exposed to psychological distress, stress, anxiety, depression and even suicidal ideations [6]. This is worsened by the fact that more health care teams have to make triage decisions and give care firstly to people with the greatest chance of short-term survival, and secondly to people with relative lack of coexisting conditions and greatest chance of long-term survival [10].

## Conclusion

The current Covid-19 pandemic has tremendous consequences on population health around the world, with various degrees of mental health repercussions. Actual strategies are mainly targeting Covid-19 patients and healthy general populations, and are chiefly based on clear and secured communication as well as continuous onsite and online psychological support by appropriates professionals. However, these strategies should be disseminated to all countries, especially sub-Saharan African ones, and approved by concerned governments. Also, adequate psychological care policies have to be developed for health care workers fighting against this pandemic. All these measures would help to maintain optimal care of Covid-19 patients and prevent deleterious mental health outcomes including suicides.

## Competing interests

All authors declare no competing interests.

## References

[1] World Health Organization (WHO) Rolling updates on Coronavirus Disease (COVID-19): events as they happen.

[2] Park SC, Park YC (2020). Mental Health Care Measures in Response to the 2019 Novel Coronavirus Outbreak in Korea. Psychiatry Investig.

[cit0003] 3Wang C, Pan R, Wan X, Tan Y, Xu L, Ho CS (2020). Immediate Psychological Responses and Associated Factors during the Initial Stage of the 2019 Coronavirus Disease (COVID-19) Epidemic among the General Population in China. Int J Environ Res Public Health.

[4] Asmundson GJG, Taylor S (2020). How health anxiety influences responses to viral outbreaks like COVID-19: What all decision-makers, health authorities, and health care professionals need to know. J Anxiety Disord.

[5] Brooks SK, Webster RK, Smith LE, Woodland L, Wessely S, Greenberg N (2020). The psychological impact of quarantine and how to reduce it: rapid review of the evidence. The Lancet.

[6] Liu S, Yang L, Zhang C, Xiang YT, Liu Z (2020). Online mental health services in China during the COVID-19 outbreak. Lancet Psychiatry.

[7] World Health Organization (WHO) (2020). Mental health and psychosocial considerations during the COVID-19 outbreak.

[8] Xiang YT, Yang Y, Li W, Zhang L, Zhang Q, Cheung T (2020). Timely mental health care for the 2019 novel coronavirus outbreak is urgently needed. Lancet Psychiatry.

[9] Jiang X, Deng L, Zhu Y, Ji H, Tao L, Liu L (2020). Psychological crisis intervention during the outbreak period of new coronavirus pneumonia from experience in Shanghai. Psychiatry Res.

[10] Rosenbaum L Facing Covid-19 in Italy-Ethics, Logistics and Therapeutics on the Epidemic´s Front Line. N Engl J Med.

